# CCR8 Expression Defines Tissue-Resident Memory T Cells in Human Skin

**DOI:** 10.4049/jimmunol.1701377

**Published:** 2018-02-02

**Authors:** Michelle L. McCully, Kristin Ladell, Robert Andrews, Rhiannon E. Jones, Kelly L. Miners, Laureline Roger, Duncan M. Baird, Mark J. Cameron, Zita M. Jessop, Iain S. Whitaker, Eleri L. Davies, David A. Price, Bernhard Moser

**Affiliations:** * Division of Infection and Immunity, Cardiff University School of Medicine, Cardiff CF14 4XN, United Kingdom;; † Systems Immunity Research Institute, Cardiff University School of Medicine, Cardiff CF14 4XN, United Kingdom;; ‡ Division of Cancer and Genetics, Cardiff University School of Medicine, Cardiff CF14 4XN, United Kingdom;; § Department of Epidemiology and Biostatistics, Case Western Reserve University, Cleveland, OH 44106;; ¶ The Welsh Centre for Burns and Plastic Surgery, Morriston Hospital, Swansea SA6 6NL, United Kingdom; and; ‖ Breast Centre, University Hospital of Llandough, Llandough CF64 2XX, United Kingdom

## Abstract

Human skin harbors two major T cell compartments of equal size that are distinguished by expression of the chemokine receptor CCR8. In vitro studies have demonstrated that CCR8 expression is regulated by TCR engagement and the skin tissue microenvironment. To extend these observations, we examined the relationship between CCR8^+^ and CCR8^−^ skin T cells in vivo. Phenotypic, functional, and transcriptomic analyses revealed that CCR8^+^ skin T cells bear all the hallmarks of resident memory T cells, including homeostatic proliferation in response to IL-7 and IL-15, surface expression of tissue localization (CD103) and retention (CD69) markers, low levels of inhibitory receptors (programmed cell death protein 1, Tim-3, LAG-3), and a lack of senescence markers (CD57, killer cell lectin-like receptor subfamily G member 1). In contrast, CCR8^−^ skin T cells are heterogeneous and comprise variable numbers of exhausted (programmed cell death protein 1^+^), senescent (CD57^+^, killer cell lectin-like receptor subfamily G member 1^+^), and effector (T-bet^hi^, Eomes^hi^) T cells. Importantly, conventional and high-throughput sequencing of expressed TCR β-chain (*TRB*) gene rearrangements showed that these CCR8-defined populations are clonotypically distinct, suggesting unique ontogenies in response to separate antigenic challenges and/or stimulatory conditions. Moreover, CCR8^+^ and CCR8^−^ skin T cells were phenotypically stable in vitro and displayed similar levels of telomere erosion, further supporting the likelihood of a nonlinear differentiation pathway. On the basis of these results, we propose that long-lived memory T cells in human skin can be defined by the expression of CCR8.

## Introduction

In steady state, the vast majority of memory T cells reside in peripheral tissues. These peripheral immune surveillance T (Tps) cells are long-lived, Ag-experienced sentinels that localize preferentially to sites of high pathogen encounter, such as the lung, gut, and skin, where they provide a first line defense against recurrent infections and control aberrant autoimmunity (1). Although originally thought to be composed entirely of effector memory T (Tem) cells migrating from the blood, it is now clear that many Tps cells exist in disequilibrium with the circulating pool (1, 2). As such, the Tps compartment incorporates both noncirculating memory T cells that persist in situ, termed tissue-resident memory T (Trm) cells, as well as memory T cells that re-enter the circulation and express tissue-specific homing receptors (3, 4). The mechanisms that regulate effector T cell trafficking during the onset of an immune response have been studied in isolator-housed mice, where the tissue T cell compartments are fully defined by the arrival of new effector T cells induced during local vaccination. Conversely, very little is known about the mechanisms that regulate the homeostatic localization and maintenance of long-lived memory T cells in peripheral tissues, although the microenvironment has been shown to direct the transformation of newly recruited effector T cells into Trm cells (5–7). It is also unclear how tissue-resident and recirculating Tps cells are generated and maintained in humans. This is an important knowledge gap in light of the fact that deregulation of the Tps network contributes to inflammatory diseases and cancer (8–11).

In contrast to murine skin, where epidermal CD8^+^CD103^+^ T cells dominate the Trm pool (2, 3), human skin is inhabited primarily by dermal CD4^+^CD103^−^ T cells (12). At present, CD69 expression remains the principal defining feature of Trm cells (12, 13). A recent study found that human CD69^+^ T cells isolated from tissues share a core gene signature with mouse Trm cells, whereas CD69^−^ T cells display features of circulating T cells (14). As such, the few skin-resident T cells that lack CD69 are thought to be either central memory T (Tcm; CCR7^+^CD62L^+^) or migratory memory T (CCR7^+^CD62L^−^) cells (12). In mice, it is clear that both Trm and Tcm cells are derived from the same effector precursors (10). However, the relationship between these distinct subsets of memory T cells that constitute the local Tps compartment in human skin requires clarification.

Our previous studies have suggested that the chemokine CCL1 controls the skin-specific localization of memory T cells expressing the chemokine receptor CCR8 (4). In humans, CCR8 is found on a large proportion of cutaneous memory T cells (∼50%), whereas very few circulating memory T cells (∼5%), all of which are cutaneous lymphocyte Ag^+^ with a Tcm or Tem phenotype, express CCR8 (15, 16). CCL1, the selective ligand for CCR8, is primarily expressed by Langerhans cells and dermal perivascular cells (15). In mice, CCR8 has been associated with the recruitment of Th2 cells to sites of atopic dermatitis (17), and CCR8 transcripts are induced in cutaneous Trm cells following the resolution of viral infections (18, 19). However, cell-surface expression of murine CCR8 was not confirmed in these studies due to a lack of Ab reagents. We showed previously that naive T cells upregulate CCR8 in the presence of soluble epidermal factors, such as keratinocyte-derived PGE_2_ and vitamin D_3_ (16, 20). These findings implicate the tissue microenvironment as a key regulator of immunological memory in the skin. In this study, we report that two distinct subsets of Tps cells can be identified in human skin on the basis of divergent phenotypic, functional, and transcriptomic profiles that cosegregate with the expression of CCR8.

## Materials and Methods

### Media

Complete AB-RPMI medium consisted of RPMI 1640 supplemented with 2 mM l-glutamine, 1% nonessential amino acids, 1% sodium pyruvate, 50 μg/ml penicillin/streptomycin, 20 mM HEPES, and 10% pooled human AB serum.

### Cell isolation

All research involving human blood and tissue samples was approved by the local Research Ethics Committee. Written informed consent was obtained from each participant in accordance with the Declaration of Helsinki. PBMCs were isolated from healthy donors via density gradient centrifugation using Lymphoprep (Axis-Shield). Human split skin samples (0.4 mm) were excised from healthy donors undergoing elective surgery using a dermatome. Sections were cut into 1 cm^2^ fragments and digested in a mixture of Dispase II (1.25 U/ml), collagenase D (1 mg/ml), and DNase I (20 U/ml) (all from Roche) for 15–30 min at 37°C. The epidermis was separated from the dermis using forceps, and both the epidermis and the dermis were subsequently cultured for 48 h at 37°C in AB-RPMI. Single-cell suspensions of migrated cells were obtained from the culture medium by passing through a 40 μm pore mesh, washed, and resuspended in AB-RPMI.

### Flow cytometry

Cells were acquired using a custom-built 20 parameter FACSAria II (BD Biosciences) and analyzed with FlowJo software (Tree Star). The commercial mAbs used in this study are listed in Supplemental Table I. Killer cell lectin-like receptor subfamily G member 1 (KLRG1)–AF488 was a kind gift from Prof. H. Pircher (University of Freiburg). Intracellular staining was performed using a FOXP3/Transcription Factor Staining Buffer Set (eBioscience). For the detection of cytokines, samples were depleted of regulatory T (Treg) cells using CD25 microbeads (Miltenyi Biotec) prior to stimulation with PMA (50 ng/ml) and ionomycin (750 ng/ml) for 6 h in the presence of brefeldin A (10 μg/ml) for the final 4 h. Streptavidin-QD605, streptavidin-BV605, or streptavidin-allophycocyanin (BD Biosciences) were used with biotinylated anti-CCR8 clones 433H or 414B (American Type Culture Collection) to detect CCR8 expression as reported previously (17). Lymphocytes were gated based on light scatter, doublets were excluded in forward scatter area/height plots, and dead cells were dumped using LIVE/DEAD Fixable Aqua (Thermo Fisher Scientific).

### Proliferation assay

Conventional CD25^−^ skin T cells were sorted by flow cytometry, labeled with CellTrace Violet (Thermo Fisher Scientific) for 10 min at room temperature, washed, and cultured for 5 d with autologous skin dendritic cells (DCs) at a ratio of 1:5 (DC/T cell) in the presence of either 50 ng/ml IL-15 or 50 ng/ml IL-7 + 50 ng/ml IL-15. As a positive control, cells were stimulated with anti-CD3/CD28 Human T-Activator Dynabeads (Thermo Fisher Scientific) at a bead/cell ratio of 1:2 in the presence of 30 U/ml IL-2.

### Transcriptome analysis

Total RNA was isolated using a Qiagen RNeasy Micro Kit (Qiagen), and cDNA was generated using a SMARTer Kit (Clontech). Libraries were prepared using a Nextera Kit (Illumina). Next-generation sequencing was performed using the Illumina HiSeq 2500 System with TruSeq Technology (Illumina). Transcriptomes were characterized via paired-end, 50 bp RNA sequencing (RNA-Seq) runs (10 samples per lane), ensuring at least 30 × 10^6^ mapped reads per sample. Analysis of gene expression was first evaluated using TopHat (21) and Cufflinks (22). A paired analysis was then performed, whereby data were mapped to the human assembly (Homo_sapiens.GRCh38) using the Burrows–Wheeler Alignment tool (23), and raw read counts per gene/transcript were assigned using featureCounts (24). Differential gene expression analysis was performed using the DESeq2 package in Bioconductor (25). Gene enrichment analysis was performed using ToppGene with a false discovery rate correction cutoff of *p* < 0.05 (26).

### Molecular analysis of TCR usage

TCR clonotyping was performed using a template-switch anchored RT-PCR (27). Amplicons were subcloned, sampled, Sanger sequenced, and analyzed as described previously (28). Assembly of TCR sequences from short-read RNA-Seq data was performed using MiXCR software (29), and postassembly repertoire analysis was performed using VDJTools (30). For repertoire overlap, similarity was measured as the clonotype-wise sum of the geometric mean frequencies and calculated as:

$$F2_{ij}=\overset{N}{\underset{k=1}{\sum }}\sqrt{\varphi_{ik}\varphi_{jk}}$$,

where $\varphi_{ik}$ and $\varphi_{jk}$ are the frequencies of clonotype *k* in samples *i* and *j*, respectively, and *N* is the total number of overlapping clonotypes.

### Single telomere length analysis

DNA was extracted from 3000 flow-sorted skin T cells using a QIAmp DNA Micro Kit (Qiagen) (31). Single telomere length analysis was carried out at the XpYp telomere as described previously (32). Briefly, 1 μM of the Telorette-2 linker was added to purified genomic DNA in a final volume of 40 μl per sample. Multiple PCRs were performed for each test DNA in 10 μl volumes incorporating 250 pg of DNA and 0.5 μM of the telomere-adjacent and Teltail primers in 75 mM Tris-HCl (pH 8.8), 20 mM (NH_4_)_2_SO_4_, 0.01% Tween-20, and 1.5 mM MgCl_2_, with 0.5 U of a 10:1 mixture of Taq (ABGene) and Pwo polymerase (Roche). DNA fragments were resolved by 0.5% Tris-acetate-EDTA agarose gel electrophoresis and identified by Southern hybridization with a random-primed α-^33^P-labeled (PerkinElmer) 5′-TTAGGG-3′ repeat probe, together with probes specific for the 1 kb (Stratagene) and 2.5 kb (Bio-Rad) markers. Hybridized fragments were detected using a Typhoon FLA 9500 Phosphorimager (GE Healthcare). The molecular sizes of the DNA fragments were calculated using a Phoretix 1D Quantifier (Nonlinear Dynamics).

### Statistics

Significance testing was performed using the Mann–Whitney *U* test, the Dunn multiple comparison test, one-way ANOVA with the Tukey posttest, and linear regression analyses in GraphPad Prism. A difference between groups was considered significant at *p* < 0.05. Heatmaps and multi-dimensional scaling analyses were generated in R.

### Accession code for RNA-Seq datasets

The RNA-Seq data reported in this manuscript are available via ArrayExpress (http://www.ebi.ac.uk/arrayexpress/experiments/E-MTAB-6370) under accession number E-MTAB-6370.

## Results

### Distribution of CCR8^+^ cells in healthy human skin

**FIGURE 1.**
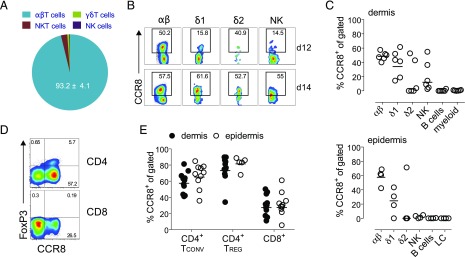
T cells and NK cells in healthy human skin express CCR8. (**A**) Pie chart showing the contribution of αβ, γδ, NKT, and NK cells to the total dermal CCR8^+^ pool. (**B**) Representative dot plots showing CCR8 expression for the indicated cell types in two donors (donor 12 [d12] and donor 14 [d14]). (**C**) Percent frequencies of αβ, γδ, NK, B, and APCs among total live dermal (top) and epidermal cells (bottom) expressing CCR8 (*n* = 4–6). LC, Langerhans cells. (**D**) Representative dot plots showing the expression of CCR8 versus FOXP3 for CD4^+^ and CD8^+^ dermal T cells. (**E**) Percent frequencies of CD4^+^FOXP3^−^ conventional T (Tconv), CD4^+^FOXP3^+^ Treg, and CD8^+^ T cells expressing CCR8 in the dermis (*n* = 10) or epidermis (*n* = 4–9). Lines denote the geometric mean.

To characterize the expression of CCR8 in healthy human skin, we separated the dermal and epidermal layers and used flow cytometry to analyze the various emigrant cell populations. αβ T cells were the most abundant immune cell type isolated from the dermal layer (44.15 ± 13.62% of total live cells; *n* = 6) and the predominant subset to express CCR8 (93.2 ± 4.1% of total CCR8^+^ emigrant skin cells; Fig. 1A) (16). In agreement with our previous report (33), γδ T cells and NK cells were also found to express CCR8, although these subsets populated the skin at much lower frequencies (0.35 ± 0.25% and 0.97 ± 0.56%, respectively) than αβ T cells (Fig. 1B, 1C). Vδ1-expressing γδ T cells, like αβ T cells, showed more consistent CCR8 expression among donors (48.73 ± 5.92% for αβ and 38.61 ± 18.54% for δ1), whereas the expression of CCR8 by NK and Vδ2-expressing γδ T cells was considerably more variable (Fig. 1B, 1C). CCR8 expression was not detected on B cells or APCs in either the dermal or epidermal layers (Fig. 1C). Among αβ T cells, CCR8 was expressed by both the CD4^+^ and CD8^+^ subsets in the dermis and epidermis (Fig. 1D, 1E), with a greater percentage of CD4^+^CCR8^+^ T cells in both compartments (59.21 ± 13.5% for dermis and 66.62 ± 15.77% for epidermis; *n* = 10; Fig. 1E). Interestingly, CD4^+^FOXP3^+^ Treg cells, which constituted ∼5–10% of dermal and epidermal CD3^+^ T cells (Fig. 1D, 1E), almost uniformly expressed CCR8 (76 ± 16.3% for dermis and 85.3 ± 2.6% for epidermis; *n* = 10, Fig. 1E). We conclude that CCR8 does not distinguish between dermal and epidermal memory T cells, whereas uniform expression on Treg cells suggests a role for CCR8 in the cellular control of skin-specific autoimmunity.

### CCR8^−^ skin T cells express genes associated with immune cell activation

In broad terms, the presence of CCR8^+^ and CCR8^−^ memory T cells in human skin could reflect either local phenotypic conversion or independent ontogenies leading to distinct tissue-resident compartments. To investigate the relationship between these subsets, we performed a whole transcriptome analysis of the CCR8^+^ and CCR8^−^ fractions sorted in parallel from the conventional CD4^+^ and CD8^+^ T cell populations of five healthy skin donors (breast, *n* = 3; thigh, *n* = 2) and three healthy blood donors (Supplemental Fig. 1). For the purpose of this study, Treg cells (CD25^hi^) were excluded from the sorted T cell subsets, and unsupervised data analysis methods were used to compare the CCR8^+^ and CCR8^−^ fractions. In a first analysis, we found that the skin replicates clustered according to tissue sampling site (breast versus thigh) for both CD4^+^ and CD8^+^ T cells (Supplemental Fig. 1A, 1B). Of note, CD8^+^CCR8^+^ T cells isolated from the thigh region displayed an intermediate pattern of expression for genes that are normally polarized between the skin (e.g., *CXCR6* and *CD69*) and the blood (e.g., *KLF2*, *S1PR1*, and *CCR10*) (Supplemental Fig. 1C). Although the relevance of this finding is presently unclear, it is important to document for future studies that the transcriptomes of skin T cells may vary according to location.

**FIGURE 2.**
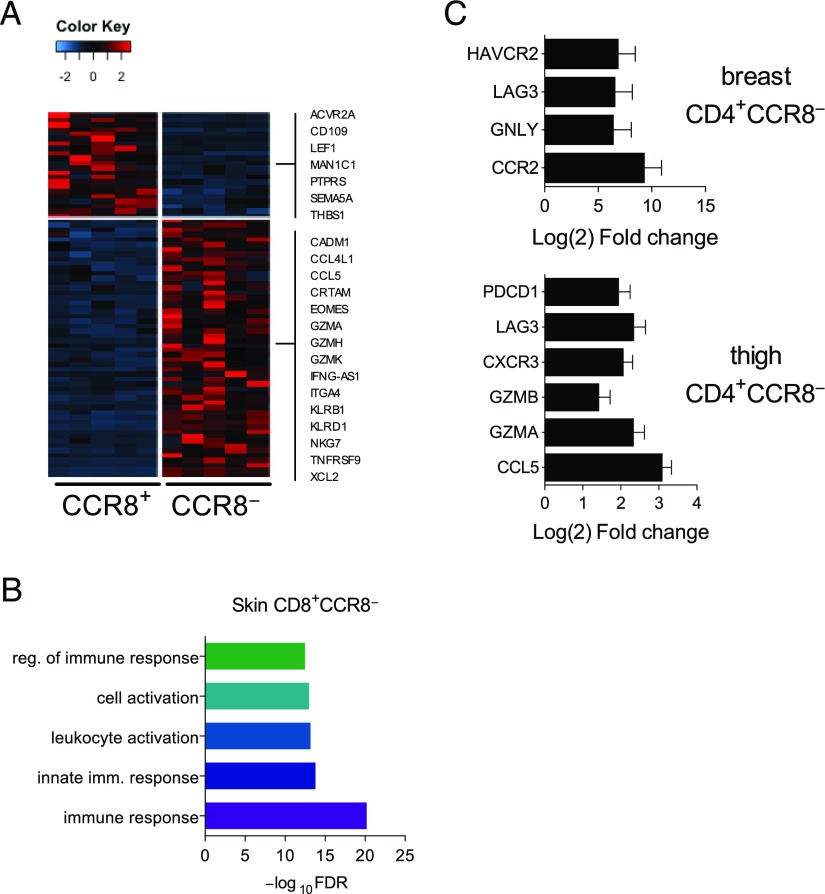
Transcriptional profiles of CCR8^+^ and CCR8^−^ skin T cells. (**A**) Heatmap of DEGs showing fold changes of >3 between skin CD8^+^CCR8^+^ and CD8^+^CCR8^−^ T cells. (**B**) Gene ontology analysis showing the top five over-represented biological processes (false discovery rate <0.05) for DEGs upregulated in CD8^+^CCR8^−^ versus CD8^+^CCR8^+^ T cells from all skin samples. (**C**) Genes associated with immune function plotted as Log2 fold change for expression in CD4^+^CCR8^−^ versus CD4^+^CCR8^+^ T cells from breast skin samples (top) and thigh skin samples (bottom).

Irrespective of sampling site, we identified 196 differentially expressed genes (DEGs) when comparing the transcriptomes of skin CD8^+^CCR8^+^ T cells versus skin CD8^+^CCR8^−^ T cells. Of these DEGs, 66 were more highly expressed in the CCR8^+^ fraction, 130 were more highly expressed in the CCR8^−^ fraction, and 13 were uniquely expressed in the CCR8^−^ fraction. Interestingly, many of the genes upregulated in the CCR8^−^ fraction encode proteins associated with immune cell activation and effector function (Fig. 2A). Further enrichment analysis confirmed this bias within the CD8^+^CCR8^−^ memory T cell subset (Fig. 2B). The upregulated genes included those encoding the cytolytic molecules perforin (*PRF1*) and granzymes (GZMs) A, B, H, and K (*GZMA*, *GZMB*, *GZMH*, and *GZMK*), the chemokines CCL3 and CCL5 (*CCL3* and *CCL5*), the chemokine receptor CXCR3 (*CXCR3*), the integrins α1 and α4 (*ITGA1* and *ITGA4*), the TNF family receptor 4-1BB (*TNFRSF9*), and eomesodermin (*EOMES*), a transcription factor that regulates effector function in T cells (34). Conversely, the CCR8^+^ fraction was enriched for expression of *LEF1*, which encodes a transcription factor involved in the generation of memory CD8^+^ T cells (Fig. 2A) (35), and genes encoding the TGF-binding protein CD109 (*CD109*), the IL-7 and IL-9 receptors (*IL-7R* and *IL-9R*), and forkhead box protein K2 (*FOXK2*). Other noteworthy genes in the CD8^+^CCR8^+^ memory T cell subset included *AKT2*, *SESN3*, and *INSR*, which encode members of the PKB/Akt signaling pathway, and *BCL9*, which encodes the transcriptional WNT/β-catenin cofactor.

The transcriptomes of skin CD4^+^ T cells were far more variable among donors and tissue sites than the transcriptomes of skin CD8^+^ T cells. However, akin to the CD8^+^ subset, more immune response genes were found to be upregulated in the CCR8^−^ fraction when samples from distinct tissue sites were analyzed independently. The CCR8^−^ fraction from breast skin samples showed greater expression of *CCR2*, *GNLY*, *LAG3*, and *HAVCR2*, which encodes TIM-3, whereas the CCR8^−^ fraction from thigh skin samples showed greater expression of several immune activation genes, including *CCL5*, *GZMA*, *GZMB*, *CXCR3*, and genes encoding the inhibitory receptors LAG3 (*LAG3*) and programmed cell death protein 1 (PD-1) (*PDCD1*) (Fig. 2C).

Overall, these data reveal that CCR8^−^ T cells in healthy skin preferentially express genes associated with immune cell activation and effector function.

### CCR8^−^ skin T cells show greater effector capacity

**FIGURE 3.**
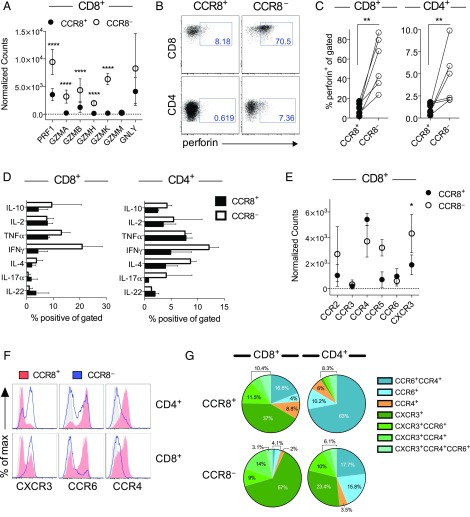
Effector phenotype and function of CCR8^−^ skin T cells. (**A**) Gene expression counts for cytolytic molecules among CD8^+^ T cells from all skin samples (*n* = 5). (**B**) Representative dot plots showing perforin expression for gated CD8^+^CCR8^+^ and CD8^+^CCR8^−^ T cells. (**C**) Percent frequencies of perforin^+^ cells within the indicated subsets of skin T cells plotted for each donor (*n* = 6). (**D**) Dermal T cells were stimulated ex vivo and analyzed for cytokine production by flow cytometry. Percent frequencies of IFNγ^+^, IL-4^+^, TNF-α^+^, IL-17a^+^, and IL-22^+^ cells among CCR8^+^ and CCR8^−^ T cells within the CD8^+^ (left) and CD4^+^ subsets (right) plotted as mean ± SD (*n* = 2). (**E**) Gene expression counts for inflammatory chemokine receptors among CD8^+^ T cells from all skin samples (*n* = 5). (**F**) Representative histograms showing the expression of CXCR3, CCR6, and CCR4 among CCR8^+^ (red) and CCR8^−^ T cells (blue) within the CD4^+^ (top) and CD8^+^ subsets (bottom). (**G**) Pie charts showing the relative distribution of chemokine receptors among the indicated subsets of skin T cells (*n* = 6). ****p < 0.0001, **p < 0.01, *p < 0.05.

The transcriptional profiles of skin T cells suggest that the CCR8^−^ fraction may be better poised to mount effector responses upon activation. Accordingly, skin CD8^+^CCR8^−^ T cells expressed higher levels of transcripts encoding cytolytic molecules compared with skin CD8^+^CCR8^+^ T cells (Fig. 3A). This finding was validated in flow cytometry experiments, which demonstrated significantly higher levels of perforin in the CCR8^−^ fraction (Fig. 3B, 3C). A similar pattern was detected among skin CD4^+^ T cells at the RNA (Fig. 2C) and protein level (Fig. 3B, 3C), although the percentage of perforin^+^ cells was much lower compared with skin CD8^+^ T cells (Fig. 3C).

To extend these functional comparisons, we measured the intracellular production of effector cytokines in response to a nonspecific activation signal. No effector cytokines were detected among untreated skin T cells (data not shown). In contrast, stimulated CCR8^+^ and CCR8^−^ skin T cells produced a variety of effector cytokines at largely equivalent frequencies, although greater proportions of CCR8^−^ T cells in both the CD4^+^ and CD8^+^ subsets expressed IFNγ (Fig. 3D).

In line with greater effector functionality, we found that CCR8^−^ T cells expressed higher levels of the inflammatory chemokine receptor CXCR3 at the RNA (Fig. 3E) and protein level (Fig. 3F). In fact, most skin CD8^+^CCR8^−^ T cells expressed CXCR3 (>80%), whereas the CCR8^+^ fraction was more likely to express CCR4 and/or CCR6 (Fig. 3E–G). Among CD4^+^ T cells, CXCR3 expression was again largely restricted to the CCR8^−^ fraction, whereas >60% of cells in the CCR8^+^ fraction coexpressed CCR4 and CCR6 (Fig. 3E–G).

Collectively, these results show that both CCR8^+^ and CCR8^−^ skin T cells can produce a full complement of effector cytokines, whereas cytotoxic potential is more firmly associated with a lack of CCR8.

### CCR8^−^ skin T cells show signs of chronic Ag exposure

**FIGURE 4.**
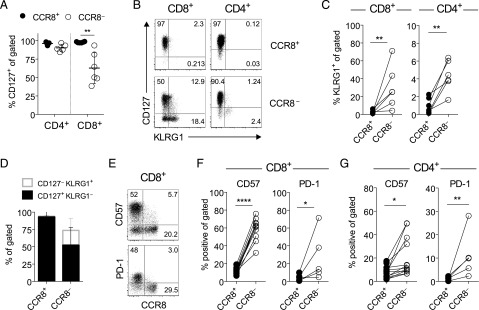
CCR8^−^ skin T cells express markers associated with chronic Ag exposure. (**A**) Percent frequencies of CCR8^+^ and CCR8^−^ T cells expressing CD127 within the CD4^+^ and CD8^+^ subsets plotted for each donor (*n* = 6). Lines denotes mean ± SD. (**B**) Representative dot plots showing CD127 versus KLRG1 expression among CCR8^+^ and CCR8^−^ T cells within the CD4^+^ and CD8^+^ subsets. (**C**) Percent frequencies of CCR8^+^ versus CCR8^−^ T cells expressing KLRG1 within the CD8^+^ (left) and CD4^+^ subsets (right) plotted for each donor (*n* = 6). (**D**) Percent frequencies of CCR8^+^ versus CCR8^−^ T cells within gated dermal CD8^+^ T cells expressing a short-lived effector memory precursor (CD127^−^KLRG1^+^; unfilled gray) or a long-lived memory precursor phenotype (CD127^+^KLRG1^−^; black) plotted as mean ± SD (*n* = 6). (**E**) Representative dot plots showing the expression of CCR8 versus CD57 (top) and PD-1 (bottom) within gated CD8^+^ T cells. (**F**) Percent frequencies of CD8^+^ T cells expressing CD57 or PD-1 within the gated CCR8^+^ versus CCR8^−^ fractions plotted for each donor (*n* = 5–11). (**G**) Percent frequencies of CD4^+^ T cells expressing CD57 or PD-1 within the gated CCR8^+^ versus CCR8^−^ fractions plotted for each donor (*n* = 5–11). ****p < 0.0001, **p < 0.01, *p < 0.05.

Local survival signals are required to ensure the long-term residency of memory T cells in peripheral tissues. IL-7 is produced in the skin under steady-state conditions and is known to play a key role in memory T cell homeostasis (36). We therefore assessed IL-7 receptor expression on skin T cells. The vast majority of CD4^+^ and CD8^+^ T cells expressed the IL-7Rα chain (CD127), most consistently within the CCR8^+^ fraction (Fig. 4A). However, the percentage of CD127^+^ cells among CD8^+^CCR8^−^ T cells varied considerably, and a significant proportion did not express CD127 (43.3 ± 16.6%, *p* = 0.0079; Fig. 4A). For murine CD8^+^ T cells, expression of CD127 in conjunction with KLRG1 has been used to define precursors of long-term memory and Trm (CD127^+^KLRG1^−^) or short-lived Tem cells (CD127^−^KLRG1^+^) (18, 37). Our analysis showed that human skin CD8^+^CCR8^+^ T cells lacked KLRG1, but uniformly expressed CD127, thereby resembling long-term memory and Trm cells (Fig. 4B–D). In contrast, the CCR8^−^ fraction incorporated a mixture of short-lived Tem cells (CD127^−^KLRG1^+^), long-term memory and Trm cells (CD127^+^KLRG1^−^), and double-negative memory T cells (Fig. 4B). The percentage of CCR8^−^ T cells with a short-lived effector memory precursor phenotype ranged from 11.6–52% within the CD8^+^ subset (Fig. 4D). A similar pattern prevailed within the CD4^+^ subset, albeit with markedly lower frequencies of KLRG1^+^ cells (Fig. 4B, 4C). Moreover, CD57 and PD-1, which are associated with terminal differentiation and exhaustion, respectively (38), were expressed almost exclusively by CD8^+^CCR8^−^ T cells (Fig. 4E, 4F) and, to a lesser extent, by CD4^+^CCR8^−^ T cells (Fig. 4G).

In peripheral blood, Tem cells have been broadly segregated into four populations based on the expression of CD27, CD28, and CD45RA (39). Cells that have undergone differentiation as a consequence of multiple rounds of division lose expression of the costimulatory receptors CD27 and CD28 and gain expression of CD45RA. In this model, early effector memory cells are defined as CD27^+/−^CD28^+^CD45RA^−^, whereas late effector memory cells lose CD28 expression (CD28^−^CD45RA^−^), and terminally differentiated effector cells re-express CD45RA (CD28^−^CD45RA^+^). The acquisition of KLRG1, CD57, and high perforin/GZM expression is also associated with progressive differentiation among peripheral blood CD8^+^ T cells (40). Despite clear differences in the expression of CD127, KLRG1, PD-1, and CD57 (Fig. 4), we found that expression of the terminal differentiation marker CD45RA in skin CD8^+^ T cells did not correlate with CCR8 expression (data not shown). The same was true for CD27 and CD28, although in some donors, a greater percentage of skin CD8^+^CCR8^−^ T cells expressed CD28 (78.5 ± 16.6% versus 58.4 ± 29% for CCR8^+^; *n* = 5). In the CD8^+^ subset, a greater percentage of CD28^−^ cells expressed CD45RA, consistent with a terminally differentiated phenotype, but the loss of CD28 did not correlate with the loss of CD127 or the acquisition of KLRG1, CD57, or PD-1 (data not shown). These findings imply either that the acquisition of markers associated with senescence and/or exhaustion is independent of effector memory differentiation in the skin or that the linear differentiation model described for blood memory T cells is not applicable to skin memory T cells.

Thus, CCR8^−^ skin T cells show more signs of previous Ag exposure (CD57^+^, KLRG1^+^, and PD-1^+^) compared with CCR8^+^ skin T cells, despite a quiescent profile in situ (CD25^−^ and Ki-67^−^; data not shown).

### CCR8^+^ skin T cells possess greater proliferative capacity

**FIGURE 5.**
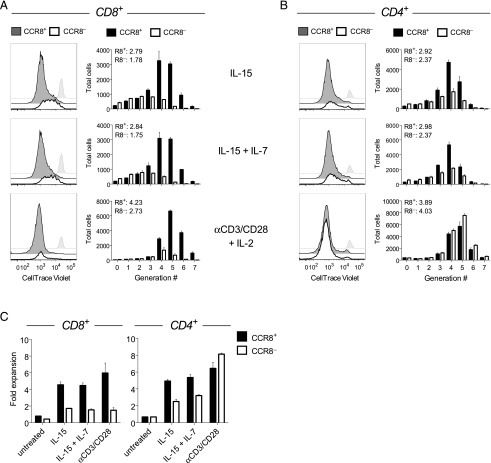
CCR8^+^ skin T cells possess greater proliferative capacity. The proliferation of flow-sorted CCR8^+^ and CCR8^−^ skin T cells in response to coculture with autologous skin DCs in the presence of IL-15 or IL-7 + IL-15, or stimulation with anti-CD3/CD28 beads in the presence of IL-2, was measured using flow cytometry to quantify the dilution of CellTrace Violet. (**A** and **B**) Representative histograms and the total number of proliferated cells for each generation of CD8^+^ (A) and CD4^+^ T cells (B). Untreated cells are denoted by gray histograms, and the proliferative index for each cell fraction is noted. (**C**) Fold expansion for CCR8^+^ and CCR8^−^ T cells within the CD8^+^ (left) and CD4^+^ subsets (right) plotted as mean ± SD (*n* = 2).

On the basis of our phenotypic and transcriptomic findings, we hypothesized that CCR8^+^ skin T cells have a proliferative or survival advantage over their CCR8^−^ counterparts, especially within the CD8^+^ subset. Previous experiments have shown that the majority of cloned skin T cells express CCR8 at variable but stable levels (15, 33). We found that CCR8^−^ T cells failed to acquire CCR8 expression upon in vitro activation, whereas CCR8^+^ T cells maintained CCR8 expression during in vitro culture (data not shown). In further experiments, we assessed the homeostatic turnover of skin T cells by culturing the flow-sorted CCR8^+^ and CCR8^−^ fractions with autologous skin DCs in the presence of either IL-15 alone or IL-7 + IL-15 (Fig. 5). CCR8^+^ skin T cells proliferated more robustly than CCR8^−^ skin T cells in the presence of IL-15, both in terms of the proliferative index (Figs. 5A, 5B) and fold expansion (Fig. 5C). The addition of IL-7 did not further enhance the proliferative response of CCR8^+^ skin T cells, despite greater expression levels of CD127 compared with CCR8^−^ skin T cells (Figs. 4A, 5A, 5B). As a control, skin T cells were also stimulated with anti-CD3/CD28 beads in the presence of IL-2. A greater number of CD8^+^CCR8^+^ T cells divided and expanded under these conditions relative to CD8^+^CCR8^−^ T cells (Fig. 5). In contrast, no significant differences were observed between CD4^+^CCR8^+^ and CD4^+^CCR8^−^ T cells, either in terms of the proliferative index (Fig. 5B) or fold expansion (Fig. 5C). These data suggest that homeostatic turnover is primarily a feature of CCR8^+^ skin T cells.

### CCR8 identifies mature resident memory T cells

**FIGURE 6.**
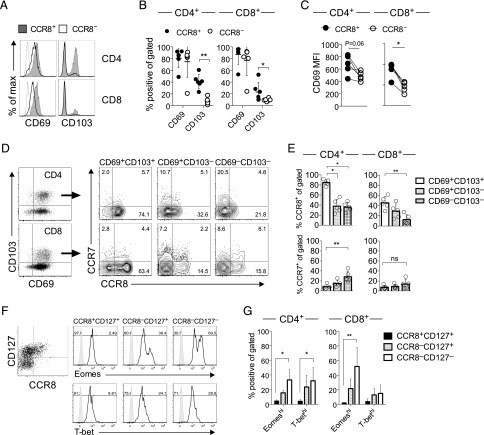
CCR8^+^ skin T cells are enriched for markers associated with resident memory. (**A**) Representative histograms showing the expression of CD69 and CD103 among CCR8^+^ and CCR8^−^ T cells within the CD4^+^ and CD8^+^ subsets. (**B**) Percent frequencies of CCR8^+^ and CCR8^−^ T cells expressing CD69 and CD103 within the CD4^+^ and CD8^+^ subsets plotted as a mean ± SD (*n* = 6–10). (**C**) Mean fluorescence intensity (MFI) values for CD69 expression among CCR8^+^ and CCR8^−^ T cells within the CD4^+^ and CD8^+^ subsets plotted for each donor (*n* = 6–10). (**D** and **E**) Expression of CCR7 and CCR8 among gated CD103^+^ Trm (CD69^+^CD103^+^; white), CD103^−^ Trm (CD69^+^CD103^−^; dotted), and non-Trm cells (CD69^−^CD103^−^; checked) within the CD4^+^ and CD8^+^ subsets depicted as representative dot plots (D) or shown as mean ± SD (*n* = 5) (E). (**F**) Representative histograms showing the expression of Eomes (top) and T-bet (bottom) among CCR8^+^CD127^+^ (black), CCR8^−^CD127^+^ (dotted), and CCR8^−^CD127^−^ (white) dermal CD8^+^ T cells. (**G**) Percent frequencies of Eomes^hi^ and T-bet^hi^ cells within the indicated subsets plotted as mean ± SD (*n* = 4). **p < 0.01, *p < 0.05.

Next, we examined whether these phenotypic and functional differences could reflect the activation state or recirculation pattern of skin T cells. The majority of conventional skin T cells isolated from the dermis lacked expression of CD25 and Ki-67, but expressed high levels of the tissue retention marker CD69 (data not shown and Fig. 6A). Although the percentage of CD69^+^ skin T cells was equivalent in the CCR8^+^ and CCR8^−^ fractions (Fig. 6B), CCR8^+^ skin T cells expressed a higher surface density of CD69 relative to CCR8^−^ T cells (Fig. 6A, 6C). Interestingly, we also found that a significantly greater percentage of CCR8^+^ dermal T cells expressed CD103 (Fig. 6A, 6B). In a recent report (12), these two markers were used to characterize the residency/recirculation potential of peripheral tissue T cells in humans. Specifically, recirculating memory T cells lacked expression of CD69, whereas CD103 distinguished two populations of Trm cells (CD69^+^CD103^+^ and CD69^+^CD103^−^). According to this definition, the vast majority of CCR8^+^ skin T cells fell within the Trm compartment, and CCR8^+^CD69^+^CD103^+^ Trm cells were more prevalent than CCR8^+^CD69^+^CD103^−^ Trm cells (Fig. 6D).

In mice, the lymph node homing receptor CCR7 is essential for the emigration of tissue T cells (41, 42). Our studies revealed that only a minority of human skin T cells express CCR7. Among the subsets defined by CD69 and CD103, non-Trm cells (CD69^−^CD103^−^) were the least abundant and contained the highest proportion of CCR7^+^ cells (Fig. 6D, 6E). Conversely, CD103^+^ Trm cells expressed the highest levels of CCR8 and the lowest levels of CCR7 (Fig. 6D, 6E).

The differentiation of murine skin CD8^+^ Trm cells depends on downregulation of the T-box transcription factors T-bet and eomesodermin (43). In agreement with our transcriptomic data (Fig. 2A), we found that the expression of T-bet and eomesodermin correlated inversely with the expression of CCR8, displaying maximal levels among CCR8^−^CD127^−^ cells within both the CD4^+^ and CD8^+^ T cell subsets (Fig. 6F, 6G).

Collectively, these data indicate that human CCR8^+^ skin T cells resemble mouse Trm cells, with a CD69^+^CD103^+^ phenotype lacking CCR7 and showing marked downregulation of T-bet and eomesodermin.

### The CCR8^+^ and CCR8^−^ fractions of skin T cells are largely unrelated

**FIGURE 7.**
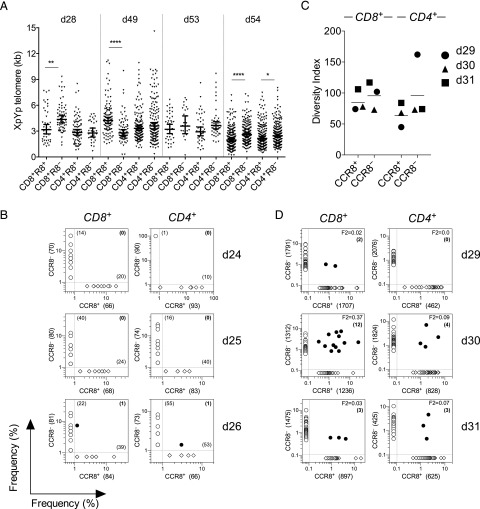
CCR8 marks distinct pools of Ag-specific memory T cells. (**A**) XpYp telomere length distributions for individual cells among the indicated subsets plotted with mean ± 95% confidence intervals. (**B**) Unique TCRβ amino acid sequences among CCR8^+^ (*x*-axis; diamond) and CCR8^−^ skin T cells (*y*-axis; open circles) within the CD4^+^ and CD8^+^ subsets plotted according to frequency with the total number of sequences denoted in brackets for each population. Shared sequences are depicted as filled circles with numbers in bold (*n* = 3). (**C** and **D**) TCRβ sequences were assembled from RNA-Seq reads using MiXCR. The estimated diversity index for each subset is plotted for each donor (*n* = 3) in (C), and the top 50 unique sequences are plotted in (D) as outlined in (B). ****p < 0.0001, **p < 0.01, *p < 0.05.

As CCR8^+^ skin T cells resemble bona fide Trm cells and CCR8^−^ skin T cells resemble effector T cells, we next asked whether CCR8^+^ T cells could differentiate into CCR8^−^ T cells following repeated cycles of activation and proliferation. For this purpose, we used single telomere length analysis (44). Telomere lengths within individual cells are defined by the activity of endogenous telomerase (terminal transferase) and the number of cell divisions, thereby providing a measure of replicative history. No consistent differences in telomere length were detected between CCR8^+^ and CCR8^−^ skin T cells within either the CD4^+^ or CD8^+^ subsets (*n* = 4; Fig. 7A), as shown recently for distinct populations of memory T cells in human peripheral blood (31). Moreover, *TERT* and *TERC*, the genes encoding telomerase, were not present on the list of DEGs (Fig. 2). These data indicate mixed proliferation histories within each subset of skin T cells, which is inconsistent with the notion of directional phenotypic interchange between the CCR8^+^ and CCR8^−^ fractions.

In peripheral blood, virus-specific memory CD8^+^ T cells often display heterogeneous phenotypes that correlate with Ag specificity. To test the idea that CCR8^+^ and CCR8^−^ skin T cells differ phenotypically as a consequence of Ag exposure rather than differentiation status, we conducted an unbiased molecular analysis of expressed *TRB* gene rearrangements in each subset using a template-switch anchored RT-PCR (45). Irrespective of CCR8 expression, skin T cells were highly polyclonal, although clonotypic diversity within the CD4^+^ subset was markedly restricted in one case (donor 24; Fig. 7B, Supplemental Fig. 2). Among all three donors tested, only one TCRβ amino acid sequence was shared between the CCR8^+^ and CCR8^−^ fractions within the CD4^+^ and CD8^+^ T cell subsets (donor 26; Fig. 7B). To extend these findings with greater coverage, we used MiXCR software to assemble CDR3β sequences from the RNA-Seq data (29). In line with our initial analysis, we found that the CCR8^+^ and CCR8^−^ fractions were polyclonal and displayed similar levels of diversity within both the CD4^+^ and CD8^+^ T cell subsets (Fig. 7B). Moreover, each repertoire was largely distinct (Fig. 7D, Supplemental Fig. 3). One exception was noted for donor 30, in whom 12 overlapping clonotypes were detected among the top 50 unique sequences within the CD8^+^ T cell compartment (F2 = 0.37). Such clonotypic overlap may reflect a phenotypic switch or divergent fates emanating from a common precursor. However, the fact that very few TCRβ sequences were shared among the different skin T cell populations within donors suggests that the CCR8^+^ and CCR8^−^ fractions arise predominantly from distinct immune activation events.

## Discussion

Tps cells in healthy human skin are composed of two memory αβ T cell compartments distinguished by CCR8 expression (15, 16). In this study, we report that CCR8^+^ skin T cells share many features of mature Trm cells, including uniform expression of CD69 with low to undetectable levels of the tissue egress receptor sphingosine-1-phosphate receptor 1 and the lymph node homing chemokine receptor CCR7. In common with Trm cells in mouse skin (43), the T-box transcriptional regulators T-bet and eomesodermin (Eomes) were also downregulated, both at the RNA and protein levels. RNA-Seq analyses revealed significantly elevated expression of *LEF1* in CD8^+^CCR8^+^ T cells (35), and a similar trend was observed for transcripts encoding Hobit (fold change = 2.6) (35, 46). Moreover, human CCR8^+^ skin T cells proliferated robustly in the presence of IL-15 and expanded in response to TCR stimulation. On the basis of these findings, we conclude that CCR8^+^ memory T cells are likely to persist long-term in healthy human skin. Of note, murine skin memory T cells also express CCR8 transcripts in response to viral infections (18, 19).

The physiological relevance of CCR8 expression is presently unclear, but it may be important for Trm cell localization to areas of chemokine production in skin tissue. CCL1, the selective ligand for CCR8, is preferentially expressed in human skin, providing support for the notion of a tissue-specific chemokine system (15). The finding that stimulated CCR8^+^ skin T cells produce a large variety of cytokines is also more compatible with a tissue-defined rather than a T cell effector type-specific homing program, as has been described for blood memory CD4^+^ T cells (47). Moreover, human skin provides essential cofactors for the induction of CCR8 (16). In particular, active vitamin D_3_ and PGE_2_ in combination with TCR signaling induce robust CCR8 expression in naive T cells during in vitro culture, suggesting that adaptive cellular immunity in the skin is governed by Ag encounter and the tissue microenvironment (20).

Unexpectedly, we found that CCR8^−^ skin T cells were phenotypically more diverse than CCR8^+^ skin T cells and functionally akin to effector T cells. Of particular note, we detected large numbers of short-lived effector memory precursor-like cells (CD127^lo^KLRG-1^+^) within the CD8^+^CCR8^−^ subset (37). Many of these cells also expressed inhibitory receptors (PD-1, Tim-3, and LAG-3) and/or markers of terminal differentiation/senescence (CD57 and KLRG1) (38). In contrast, CD8^+^CCR8^+^ T cells expressed low levels of inhibitory receptors, CD57, and KLRG1, and uniformly expressed CD127. The CCR8^−^ pool was further enriched for expression of the inflammatory chemokine receptors CXCR3, CCR2, and CCR5, and higher frequencies of CCR8^−^ skin T cells produced effector cytokines in response to stimulation compared with CCR8^+^ skin T cells, potentially reflecting greater expression of the T-box transcription factors T-bet and Eomes and relatively low level expression of *LEF1* (34, 35). Not surprisingly, the CCR8^−^ fraction of skin CD8^+^ T cells was also enriched for expression of cytolytic molecules as well as integrin α1 (CD49a), consistent with a recent report (48). Moreover, CCR8^−^ skin T cells showed defective proliferation and survival relative to CCR8^+^ skin T cells in response to homeostatic cytokines or triggering via the TCR. In sum, these data reveal a striking degree of heterogeneity among CCR8^−^ skin T cells, many of which display an end-stage effector phenotype, in contrast to the uniform memory characteristics of CCR8^+^ skin T cells.

On the basis of these striking phenotypic and functional differences, we hypothesized that the CCR8^−^ effector cell fraction derives from the CCR8^+^ memory fraction in response to local reactivation and/or differentiation. However, we detected no significant differences between the CCR8-defined subsets with respect to telomere length, telomerase gene transcription, or the expression of CD27, CD28, and CD45RA. Moreover, CCR8^+^ skin T cells retained CCR8 during in vitro culture, whereas CCR8^−^ skin T cells failed to upregulate CCR8 in response to ex vivo stimulation (data not shown), even in the presence of epidermis-conditioned medium (16). A linear relationship between these two compartments therefore seems unlikely, the data instead suggesting a stable memory phenotype in human skin defined by the expression of CCR8.

A recent vaccination study in humans indicated that a single naive precursor can give rise to functionally diverse effector T cells with shared Ag specificity (49). It has also been shown that distinct memory T cell compartments (Trm and Tcm) can be generated in response to challenge with several experimental Ags in a mouse model of cutaneous vaccination (10). Similarly, two phenotypically distinct pools of Trm cells with shared pathogen specificity can be generated in the mouse gut and salivary glands (50, 51). In contrast to these studies, our molecular analysis of expressed *TRB* gene rearrangements revealed profound discrepancies between the CCR8-defined compartments, suggesting that CCR8^+^ and CCR8^−^ skin T cells recognize distinct Ags. Of note, several overlapping clonotypes were detected in one donor, but the degree of commonality was minimal compared with that reported for experimentally induced mouse skin Trm and blood Tcm cells (10). Instead, our findings are reminiscent of another study in which human skin Treg cells and peripheral blood Tcm cells were found to be clonotypically distinct, thereby suggesting that Ag-driven differentiation of tissue Treg cells does not concomitantly induce circulating memory T cells with corresponding specificities (52).

In humans, a population of CD103^−^ Trm cells with high levels of cytolytic activity and inhibitory receptor expression has recently been identified in the lung, although the origin of these cells is presently unclear (53). There is substantial evidence from mouse vaccination studies that Trm cells develop locally from effector cells activated in tissue-draining secondary lymphoid tissues (2). Moreover, it is known that the tissue microenvironment instructs effector T cell homing and plays an important role in promoting Trm differentiation (6, 54). As a consequence, vaccines should be administered directly at the site of vulnerability to elicit long-lasting immune protection. Interestingly, certain routes of vaccination generate effector T cell populations with broader tissue-homing capabilities, enabling them to enter peripheral tissues that are unrelated to the original site of vaccination (55). In support of this notion, a recent study in mice revealed that distal vaccination induced a CXCR3^hi^ Trm cell population in the lung, whereas local intranasal challenge promoted the differentiation of a more protective CXCR3^lo^ Trm cell population (56). The route of immunization therefore impacts on the quality of memory T cells that become lodged in the target organ, further underscoring the importance of the tissue microenvironment and draining lymph nodes in the generation of local protective immunity.

Collectively, the experimental data reported thus far support the view that Trm cells develop from recruited effector T cells. In contrast, our results indicate the presence of a more complex memory T cell compartment in healthy human skin. As naive T cells require a TCR-mediated signal in the presence of skin-derived soluble factors to upregulate CCR8 (16, 20), we propose that long-lasting immune protection against locally encountered Ags depends on the induction of skin-resident CCR8^+^ memory T cells. Moreover, we suggest that the efficacy of skin-targeted vaccines may be enhanced by strategies designed to maximize the in situ induction of Ag-specific T cells expressing CCR8.

## Supplementary Material

Data Supplement
