# Supplementary material for: CCR8 Expression Defines Tissue-Resident Memory T Cells in Human Skin
Source: J Immunol. 2018 Feb 2;200(5):1639–50. doi: 10.4049/jimmunol.1701377 (PMC5818732; doi:10.4049/jimmunol.1701377)
Supplement: Data Supplement [file JI_1701377.zip › JI_1701377_Supplemental_Material_1.pdf]

Table S1. Commercial antibodies used for immunostaining.

Antigen	Clone	Fluorochrome	Supplier
CD3	OKT3	BV785	BioLegend
CD3	UCHT1	Pacific Blue	Becton Dickinson
CD4	S3.5	PE-Cy5.5	Caltag
CD8	RPA-T8	BV711	BioLegend
CD14	M5E2	Pacific Blue	Becton Dickinson
CD19	HIB19	PE-Cy5	Becton Dickinson
CD25	M-A251	APC-H7	Becton Dickinson
CD27	1A4LDG5	PE-Cy5	Coulter
CD28	CD28.2	BV421	BioLegend
CD45RA	2H4LDH11LDB9	ECD	Beckman Coulter
CD45RA	HI100	APC	Becton Dickinson
CD45RO	UCHL1	ECD	Beckman Coulter
CD56	B159	PE-Cy7	Becton Dickinson
CD57	HNK-1	FITC / PE	Becton Dickinson
CD69	FN50	FITC	Becton Dickinson
CD103	Ber-ACT8	PE	Becton Dickinson
CD127	A019D5	PE / BV421	BioLegend
CCR4	1G1	PE-Cy7	Becton Dickinson
CCR6	11A9	PE	Becton Dickinson
CCR7	3D12	PE-Cy7	Becton Dickinson
CCR10	314305	APC	R&D Systems
CXCR3	49801.111	FITC	R&D Systems
Perforin	dG9	APC	BioLegend
PD-1	MIH4	APC	Becton Dickinson
Ki-67	B56	PE	Becton Dickinson
Eomes	WD1928	PE	eBioscience
T-bet	4B10	PE	eBioscience
FoxP3	236A/E7	AF700	eBioscience
IFNγ	B27	FITC	Becton Dickinson
IL-4	8D4	PE	Becton Dickinson
IL-10	JES3-9D7	PE	eBioscience
IL-2	5344.111	FITC	Becton Dickinson
IL-17	64DEC17	AF488	eBioscience
IL-22	142928	PE	R&D Systems
TNFα	IPM2	PE	Beckman Coulter

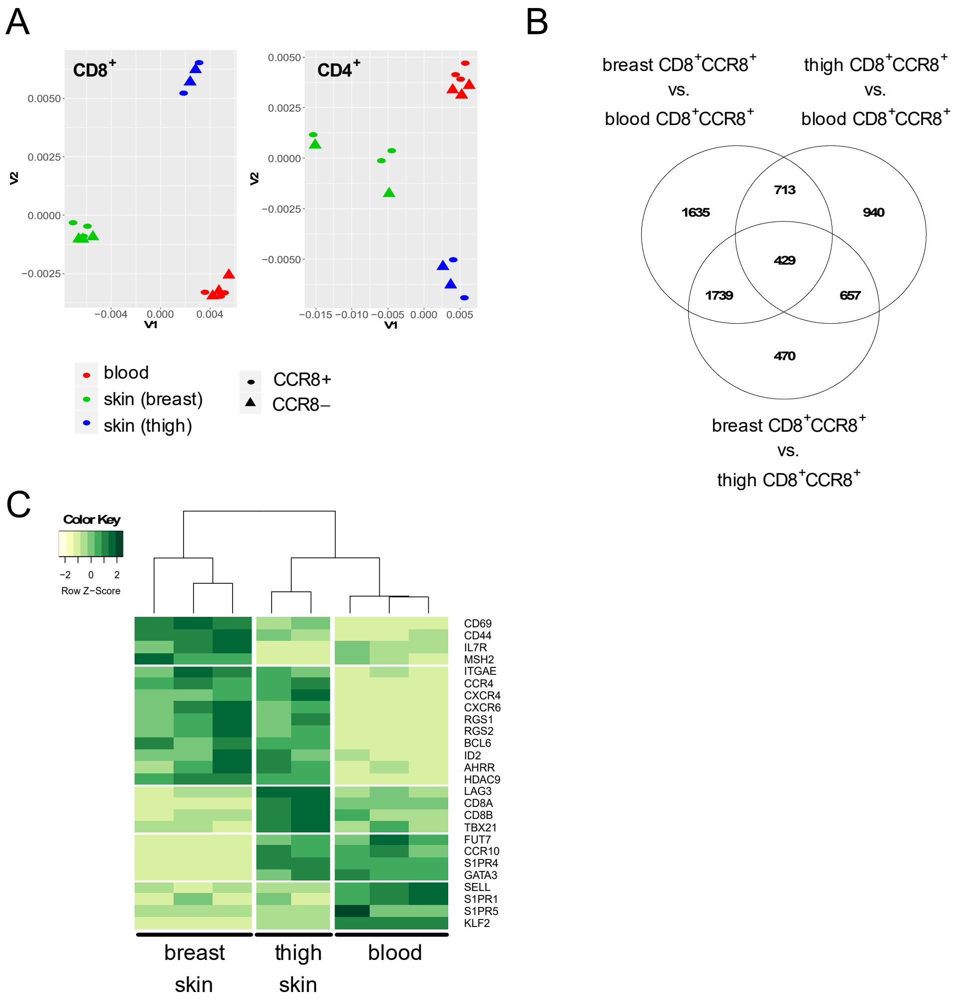

Figure S1. Analysis of RNA-Seq data. (A) Multi-Dimensional Scaling analysis of replicate CCR8$^+$ (circles) and CCR8$^-$ T cell transcriptomes (triangles) within the CD8$^+$ (top) and CD4$^+$ subsets (bottom) isolated from breast skin, thigh skin, and peripheral blood. The distance between samples indicates the average linkage difference between subsets. **(B)** Venn diagram showing the overlap of DEGs from three independent comparisons of CD8$^+$CCR8$^+$ T cells isolated from breast skin, thigh skin, and peripheral blood. **(C)** Heatmap of genes that normally display biased expression in skin memory T cells *versus* blood memory T cells, showing data for CD8$^+$CCR8$^+$ T cells isolated from breast skin, thigh skin, and peripheral blood.

d24

CD8+CCR8+

TRBV	CDR3	TRBJ	Freq (%)
28	CASSPPGGHSNTGELF	2-2	19.7
12-3	CASSLAGEKLF	1-4	12.1
7-2	CASSLGSRGIDNEQF	2-1	10.6
20-1	CSALNPGGSNQPQH	1-5	7.6
9	CASSADGDSNQPQH	1-5	6.1
5-6	CASSLGSSYEQY	2-7	4.5
5-1	CASSPTGTGDVTF	1-1	4.5
3-1	CASSQDFKRVSQQY	2-7	4.5
3-1	CASSQTGDPTEAF	1-1	4.5
28	CASSPPGGHSNTGELV	2-2	3.0
28	CASSFGLSYEQY	2-7	3.0
15-2	CATSRDHAVWGEQY	2-7	3.0
12-3	CASSLGGIHNEQF	2-1	3.0
7-8	CASGRTGNTEAF	1-1	3.0
7-2	CASSLSPGTANEQF	2-1	3.0

CD8+CCR8-

TRBV	CDR3	TRBJ	Freq (%)
2	CASGIPRGYT	1-2	30.0
12-3	CASSFTGFSSYNEQF	2-1	15.7
4-1	CASSLWTGGSYEQY	2-7	10.0
2	CASSEDTSTNEKLF	1-4	7.1
29-1	CSVRAQYSYEQY	2-7	5.7
28	CASSFGADTQY	2-3	5.7
28	CASSRPGGQF	2-1	5.7
29-1	CSVEDDSSYEQY	2-7	4.3
28	CASSRQGSETQY	2-5	4.3
20-1	CSASSLADTYEQY	2-7	4.3
13	CASSLAGSYNEQF	2-1	2.9

CD4+CCR8+

TRBV	CDR3	TRBJ	Freq (%)
7-2	CASRSRGRGEKLF	1-4	36.6
13	CASSLEGLVSPEQF	2-1	22.6
7-8	CASSGRQGTDYGYT	1-2	15.1
7-9	CASKDGQGHFPGELF	2-2	14.0
21-1	CASSNGLLGLAGVNTGELF	2-2	6.5

CD4+CCR8-

TRBV	CDR3	TRBJ	Freq (%)
5-1	CASSLAGLDYT	1-2	100.0

d25

CD8+CCR8+

TRBV	CDR3	TRBJ	Freq (%)
7-3	CASSLGQGGYNEQF	2-1	11.8
27	CASSYIARVYEQY	2-7	8.8
12-4	CASRRLAGGPYEQF	2-1	8.8
30	CAWSLGRADTQY	2-3	7.4
9	CASSPGEWWSTEAF	1-1	7.4
30	CAWGSTLEAF	1-1	5.9
12-3	CASSSDTRAYEQY	2-7	5.9
5-1	CASSSESYYNEQF	2-1	5.9
28	CASSSYGTYRGDTQY	2-3	4.4
7-9	CASSQTGQGPYEQY	2-7	4.4
20-1	CSASPPISSYNEQF	2-1	2.9
12-3	CASSPRTGAGEQF	2-1	2.9
11-2	CASSLGPGAYEQY	2-7	2.9
11-2	CASSSGLAAGELF	2-2	2.9
9	CASSATGTSGANEQF	2-1	2.9
7-3	CASSLGGGYNEQF	2-1	2.9

CD8+CCR8-

TRBV	CDR3	TRBJ	Freq (%)
7-2	CASSPWDGTSSYNEQF	2-1	12.5
12-4	CASSLGGTGEKLF	1-4	10.0
27	CASSLSGGGNQPQH	1-5	5.0
24-1	CATSDPRAAGLAGPNTGELF	2-2	5.0
7-2	CASSLTNSYEQY	2-7	5.0
20-1	CSARDHRARAYYGYT	1-2	3.8
10-1	CASMVKALSLSYEQY	2-7	3.8
30	CAWNGLNYGYT	1-2	2.5
28	CASRGQGYEQY	2-7	2.5
18	CASSPEGDRGIGEQY	2-7	2.5
12-4	CASSPYSGGSTDTQY	2-3	2.5
12-3	CASSAGPNTEAF	1-1	2.5
7-6	CASSPLAGGPLNSYNEQF	2-1	2.5
7-6	CASSLAGEVNEQF	2-1	2.5
6-5	CASSRDRDLWEQY	2-7	2.5
6-2/6-3	CASSYWRLEAVEETQY	2-5	2.5
6-2/6-3	CASSYSGQGLYEQY	2-7	2.5
3-1	CASSPLVGGAENEQF	2-1	2.5

CD4+CCR8+

TRBV	CDR3	TRBJ	Freq (%)
6-1	CASRDKGTGLFEQY	2-7	7.2
3-1	CASSLRDRRKTGELF	2-2	6.0
30	CAWSLGGELF	2-2	4.8
28	CASRRTGRRNIQY	2-4	4.8
20-1	CRAWGRPNTEAF	1-1	4.8
20-1	CSARKTGSTDTQY	2-3	4.8
7-9	CASSLAGWRVSWHEQF	2-1	4.8
7-2	CASSLDSYEQY	2-7	4.8
6-4	CASSDSSIKAGATGELF	2-2	4.8
12-3	CASRRNPGQGPLDFYGYT	1-2	3.6
10-3	CAISRGGSPDTQY	2-3	3.6
6-6	CASSPLLGRAY	2-3	3.6
27	CASRGTGGAGNNSPLH	1-6	2.4
19	CASSDQVRETQY	2-5	2.4
7-8	CASSTRTRSPGAPHNEQF	2-1	2.4
7-8	CASSLNVGEIGELF	2-2	2.4
7-2	CASSFKGQGTEQY	2-7	2.4
6-5	CASSYSPVGSSPLH	1-6	2.4
5-1	CASSLNPGPTNEKLF	1-4	2.4

CD4+CCR8-

TRBV	CDR3	TRBJ	Freq (%)
11-3	CASSPGRSDEQF	2-1	23.0
7-7	CASSPGDLNTGELF	2-2	20.3
29-1	CSTLRGGADNEQF	2-1	16.2
19	CAIAARGDQPQH	1-5	6.8
12-3	CASSLDGMAGELF	2-2	5.4
11-2	CASSLGVRDWETQY	2-5	5.4
20-1	CSASLGPTWGNQPQH	1-5	4.1
6-6	CASSPMGTGETEAF	1-1	4.1
28	CAHGTYNEQF	2-1	2.7
12-3	CASSLGGGSPDTQY	2-3	2.7
5-5	CASSQGYRGSNEQF	2-1	2.7

d26

CD8+CCR8+

TRBV	CDR3	TRBJ	Freq (%)
4-1	CASSQGGGLAGGSSYNEQF	2-1	17.9
6-1	CASSEAKGLNTEAF	1-1	4.8
5-1	CASSYDSYEQY	2-7	4.8
4-1	CASSPSGGASNEQF	2-1	4.8
19	CASTTERQGGDTEAF	1-1	4.8
7-6	CASSLGTGTVSYEQY	2-7	3.6
6-5	CASSDDRAGLSEKLF	1-4	3.6
6-5	CASSTPLDRVNEQF	2-1	3.6
2	CASREPGMSMAKNIQY	2-4	3.6
2	CASSAGGLAAYNEQF	2-1	3.6
20-1	CRAYHVGVYGYT	1-2	2.4
14	CASSPLGGHRLGANVLT	2-6	2.4
13	CASSVRGPLAGARNEQF	2-1	2.4
9	CASSVVSGGSTDTQY	2-3	2.4
7-6	CASSLNPSTDTQY	2-3	2.4
6-2	CASSCCHGHTEAF	1-1	2.4
4-1	CASSQGGGPAGGSSYNEQF	2-1	2.4
2	CASSGGGTGPDSWTDSPLH	1-6	2.4
2	CASSEGRQVYEQY	2-7	2.4

CD8+CCR8-

TRBV	CDR3	TRBJ	Freq (%)
4-1	CASSQDGVSGPQH	1-5	18.5
4-3	CASSQASGGPTDTQY	2-3	14.8
6-5	CASTNYLGGGHQDTEAF	1-1	7.4
**6-5**	**CASSASEFREQY**	**2-7**	**7.4**
11-1	CASGNEQF	2-1	6.2
12-4	CASSLAPGQGLSYEQY	2-7	4.9
7-9	CASSLGNTEAF	1-1	4.9
6-5	CASSYSGQINEKLF	1-4	4.9
5-1	CASSLGAGTNYEQY	2-7	4.9
4-2	CASSPQAFNSPLH	1-6	4.9
19	CASKGSGEEQY	2-7	3.7
6-2/6-3	CASSYSRNEQF	2-1	3.7
5-1	CASSLDLGMNTEAF	1-1	2.5

CD4+CCR8+

TRBV	CDR3	TRBJ	Freq (%)
11-2	CASSLTSGGASYEQY	2-7	6.1
5-1	CASSSGQPNTEAF	1-1	4.5
7-2	CASSPRPGSEQY	2-7	4.5
**7-2**	**CASSLTAGTDTQY**	**2-3**	**3.0**
7-9	CASSLGGYSNQPQH	1-5	3.0
13	CASSRSTGESSDEQY	2-7	3.0
20-1	CSARDSGSYNEQF	2-1	3.0
20-1	CSARTGGLQETQY	2-5	3.0
20-1	CSARDHINRYT	1-2	3.0

CD4+CCR8-

TRBV	CDR3	TRBJ	Freq (%)
27	CASSPLDSNTEAF	1-1	8.2
5-1	CASNDNAGVNTEAF	1-1	6.8
27	CASSPSTVWSEKLF	1-4	4.1
2	CAGARDRVQHEQY	2-7	2.7
7-2	CASSLEGQGAAEAF	1-1	2.7
7-2	CASSAEISYNEQF	2-1	2.7
12-3	CASSLEGGLAGGIGEQ	2-1	2.7
12-4	CASRHAGNQPQH	1-5	2.7
28	CASSTRRNNQPQH	1-5	2.7
30	CAWSVSSELEQY	2-7	2.7

Figure S2. Conventional analysis of expressed *TRB* gene rearrangements. The most frequent clonotypes (frequency >2%) are listed for the indicated subsets. Clonotypes appearing in bold represent sequences found within the CCR8^{+} and CCR8^{-} populations.

CD8+CCR8+

d29

TRBV	CDR3	TRBJ	Count	Freq (%)
12-3	CASSIAYEQYF	2-7	380	22.3
2	CASSEGLGAATDTQYF	3-3	352	20.6
18	CASSPTSGANVLTF	2-6	74	4.3
7-9	CASSSGEAFF	1-1	69	4.0
27	CASSSNEQYF	2-7	63	3.7
2	CASTRDRNTGELFF	2-2	52	3.0
5-1	CASSFGGANEQFF	2-1	35	2.1
**27**	**CASSPGPWRDYYGYTF**	**1-2**	**34**	**2.0**
18	CASSPSFLYGYTF	1-2	35	2.1
20-1	CSARDAWETTNEQFF	2-1	34	2.0
7-9	CASSFNSYEQYF	2-7	30	1.8
12-3	CASSPLAGGSDTQYF	2-3	22	1.3
30	CASWGTTDSGANVLTF	2-6	22	1.3
5-6	CASSFPGENQPQHF	1-5	20	1.2
5-1	CASSFGASGYTF	1-2	17	1.0

d30

TRBV	CDR3	TRBJ	Count	Freq (%)
**6-3**	**CASRRRGEGTEAFF**	**1-1**	**313**	**25.3**
6-3	CASSRRALPSNEQFF	2-1	178	14.4
**9**	**CASSPPGQGFYNEQFF**	**2-1**	**68**	**5.5**
**15**	**CATSRVGITGNTIYF**	**1-3**	**47**	**3.8**
**11-2**	**CASTQQGGDTEAFF**	**1-1**	**37**	**3.0**
**7-3**	**CASSLYENTEAFF**	**1-1**	**37**	**3.0**
2	CATEPETTYEQYF	2-7	33	2.7
**7-9**	**CASSLRYEQYF**	**2-7**	**28**	**2.3**
**27**	**CATRQNTEAFF**	**1-1**	**26**	**2.1**
5-1	CASSFEGWDSEAFF	1-1	25	2.0
**7-6**	**CASSLADRELFF**	**2-2**	**20**	**1.6**
15	CATSSKSTDTQYF	2-3	19	1.5
9	CASSVAYRGGEAFF	1-1	17	1.4
**7-9**	**CASPSGGNTGELFF**	**2-2**	**16**	**1.3**
21-1	CASSPRQYYEQYF	2-7	14	1.1

d31

TRBV	CDR3	TRBJ	Count	Freq (%)
19	CASSPMTGSYNEQFF	2-1	179	20.0
27	CASQRLGGNTIYF	1-3	106	11.8
**9**	**CASSVAYRGREQFF**	**2-1**	**38**	**4.2**
**6-5**	**CASSTGVMNTEAFF**	**1-1**	**19**	**2.1**
5-4	CASSFDRGPYGYTF	1-2	19	2.1
4-1	CASSPSQERPILDTQYF	2-3	16	1.8
18	CASSPGDRANDEQFF	2-1	15	1.7
12-4	CASSLGRVGSYNSPLHF	1-6	14	1.6
25-1	CASSEWGTGHTEAFF	1-1	14	1.6
12-4	CASSLGTAWGYTF	1-2	14	1.6
6-5	CASSYTGGTLDTQYF	2-3	13	1.4
5-6	CASSLAQAGSAPLHF	1-6	12	1.3
6-2	CASTRLGVEAFF	1-1	12	1.3
19	CASSIVNNQPQHF	1-5	10	1.1
29-1	CSVDTGMNTEAFF	1-1	10	1.1

CD8+CCR8-

d29

TRBV	CDR3	TRBJ	Count	Freq (%)
20-1	CSARPLHTYEQYF	2-7	163	9.1
12-3	CASSLAGRGDTQYF	2-3	158	8.8
7-8	CASSQGGTYEQYF	2-7	102	5.7
20-1	CSARDRASYNEQFF	2-1	70	3.9
7-9	CASSLAGTDTQYF	2-3	67	3.7
5-1	CASSLEEGGNTIYF	1-3	49	2.7
6-6	CASSYDSGELFF	2-2	48	2.7
7-9	CASSLDRSPLHF	1-6	42	2.3
28	CASSLRGTKTQYF	2-5	32	1.8
3-1	CASSQLKRGTRSYNEQFF	2-1	32	1.8
4-1	CASSSGQTNQPQHF	1-5	28	1.6
7-9	CASRGTKDRGTYGYTF	1-2	25	1.4
6-5	CASSYGTFGYTF	1-2	24	1.3
6-5	CASAQGLNTGELFF	2-2	23	1.3
7-2	CASSQYPGQKYF	2-3	22	1.2

d30

TRBV	CDR3	TRBJ	Count	Freq (%)
**15**	**CATSRVGITGNTIYF**	**1-3**	**97**	**7.4**
**7-9**	**CASSLRYEQYF**	**2-7**	**88**	**6.7**
**10-3**	**CAIRDSPYNSPLHF**	**1-6**	**76**	**5.8**
**27**	**CASARLTEDSNQPQHF**	**1-5**	**69**	**5.3**
27	CASRRNTEAFF	1-1	60	4.6
**7-3**	**CASSLYENTEAFF**	**1-1**	**56**	**4.3**
7-8	CASSPRSGGLPSYEQYF	2-7	37	2.8
27	CASSPGTGGWSGYTF	1-2	36	2.7
**7-9**	**CASTTSGRAGEQYF**	**2-7**	**31**	**2.4**
30	CAWSVSGSLELFF	2-2	30	2.3
29-1	CSVEGGGLDGYTF	1-2	30	2.3
**6-3**	**CASRRRGEGTEAFF**	**1-1**	**29**	**2.2**
**9**	**CASSPPGQGFYNEQFF**	**2-1**	**27**	**2.1**
2	CASSQTGDGYTF	1-2	25	1.9
**7-6**	**CASSLADRELFF**	**2-2**	**21**	**1.6**

d31

TRBV	CDR3	TRBJ	Count	Freq (%)
7-8	CASIGQENTGELFF	2-2	148	10.0
4-2	CASSQGESSYNEQFF	2-1	73	4.9
27	CASSLFGASTDTQYF	2-3	64	4.3
12-4	CASSSLGQYNEQFF	2-1	60	4.1
12-3	CASSLAGQGSQPQHF	1-5	47	3.2
7-9	CASSTSEGLNTEAFF	1-1	45	3.1
2	CASMGIPRNEQFF	2-1	42	2.8
11-2	CASSLAPGSIAFF	1-1	41	2.8
29-1	CSVEDGDRTAGYTF	1-2	36	2.4
4-1	CASSPQGEGQPQHF	1-5	34	2.3
28	CASSLGGGNTEAFF	1-1	27	1.8
7-8	CASEGGGALLSTDTQYF	2-3	24	1.6
24-1	CATSDRWPIPGELFF	2-2	21	1.4
19	CASSIGMATEAFF	1-1	21	1.4
6-3	CASSPTSHNEQFF	2-1	20	1.4

CD4+CCR8+

d29

TRBV	CDR3	TRBJ	Count	Freq (%)
29-1	CSVDSRTGADTQYF	2-3	35	7.6
5-1	CASSEGTEYNEQFF	2-1	31	6.7
2	CASSVSGSYEQYF	2-7	32	6.9
10-3	CAIRDSSGNTIYF	1-3	25	5.4
7-2	CASSLGRSQGTRPQHF	1-5	24	5.2
7-9	CASNVGGSNTEAFF	1-1	20	4.3
4-3	CASSHGLAEETQYF	2-5	19	4.1
20-1	CSARDGTASTDTQYF	2-3	17	3.7
12-4	CASSSQGMEQFF	2-1	16	3.5
7-2	CASSLSGGFSNQPQHF	1-5	15	3.2
9	CASSVAGTVIYEQYF	2-7	15	3.2
20-1	CSARDVLGSGSSYEQYF	2-7	14	3.0
15	CATSRVAGLLGTIYF	1-3	14	3.0
20-1	CSAPDLSGSPLHF	1-6	14	3.0
7-9	CASSPSYEQYF	2-7	14	3.0

d30

TRBV	CDR3	TRBJ	Count	Freq (%)
**30**	**CAWTGTRGYTF**	**1-2**	**44**	**5.3**
2	CASRWDKNTEAFF	1-1	44	5.3
3-1	CASSQDLGIRNQPQHF	1-5	42	5.1
29-1	CSVDTSTDTQYF	2-3	34	4.1
6-5	CASSYRPDYGYTF	1-2	33	4.0
29-1	CSVQGLRDGYTF	1-2	31	3.7
28	CASSSPLTEAFF	1-1	29	3.5
5-4	CASSRDSNEQFF	2-1	27	3.3
7-9	CASSSKWDGANVLTF	2-6	25	3.0
2	CASSEMTAEAFF	1-1	22	2.7
10-3	CAISEQGGKAGELFF	2-2	21	2.5
7-2	CASSLEGVSGRHYNEQFF	2-1	20	2.4
25-1	CASTLGTGGYNSPLHF	1-6	18	2.2
5-4	CASSASGLASNEQFF	2-1	18	2.2
4-2	CASSQGTSGRYNEQFF	2-1	17	2.1

d31

TRBV	CDR3	TRBJ	Count	Freq (%)
10-3	CAIGPDHEQYF	2-7	57	9.1
5-4	CASSLAPGLEGFF	2-1	43	6.9
12-4	CASSFSGLGKLFF	1-4	24	3.8
27	CASSFGDRGRSGYTF	1-2	23	3.7
12-4	CASSLAGRGRRNTEAFF	1-1	21	3.4
12-3	CASSLGSWLGVNTEAFF	1-1	19	3.0
7-2	CASSQQGARETQYF	2-5	19	3.0
7-6	CASSLRRRVSGGELFF	2-2	16	2.6
5-6	CASSRTGKKNIQYF	2-4	15	2.4
6-2	CASRTRQGNQPQHF	1-5	14	2.2
12-3	CASSLSQEGRAFF	1-1	14	2.2
12-3	CASRRQEGYGYTF	1-2	14	2.2
2	CASSTLYNEQFF	2-1	14	2.2
7-2	CASSLRAGGSVYNEQFF	2-1	13	2.1
19	CASSIRAPRPEQFF	2-1	13	2.1

CD4+CCR8-

d29

TRBV	CDR3	TRBJ	Count	Freq (%)
7-9	CASSGTTGGYEQYF	2-7	50	2.4
29-1	CSASRVLNTEAFF	1-1	47	2.3
27	CASSLFRGGQPQHF	1-5	48	2.3
6-5	CASSGSFGDTQYF	2-3	37	1.8
7-3	CASKGQMNTEAFF	1-1	38	1.8
5-1	CASSFTGTTSGELFF	2-2	37	1.8
7-2	CASSFVGLTDTQYF	2-3	32	1.5
12-3	CASSLGLGGTGELFF	2-2	34	1.6
3-2	CASSSGATYEQYF	2-7	29	1.4
5-1	CASSQVNTEAFF	1-1	27	1.3
5-5	CASSLLSEAFF	1-1	26	1.3
12-4	CASSYLNTEAFF	1-1	24	1.2
10-3	CATGLAGEDTQYF	2-3	23	1.1
12-4	CASIGQGLTEAFF	1-1	23	1.1
6-1	CASSEEQGNTIYF	1-3	21	1.0

d30

TRBV	CDR3	TRBJ	Count	Freq (%)
6-5	CASSPTGVGYTF	1-2	215	11.8
**20-1**	**CSAPVAGDGEQYF**	**2-7**	**128**	**7.0**
7-9	CASSRLDTQYF	2-3	104	5.7
12-3	CASRSGGYEQYF	2-7	67	3.7
29-1	CSVDGGYAGELFF	2-2	51	2.8
6-5	CASRPTGYYGYTF	1-2	51	2.8
7-2	CASSQGGQGTYGYTF	1-2	42	2.3
7-2	CASSYPGLASGELFF	2-2	41	2.2
24-1	CATSDGPRETQYF	2-5	41	2.2
**30**	**CAWTGTRGYTF**	**1-2**	**40**	**2.2**
19	CASSRTQGSSAMNTEAFF	1-1	40	2.2
25-1	CASTGQGGIYEQYF	2-7	36	2.0
15	CATSREAGETQYF	2-5	36	2.0
12-3	CASSREDARVQFF	2-1	35	1.9
7-2	CASSLARGGLYNEQFF	2-1	32	1.8

d31

TRBV	CDR3	TRBJ	Count	Freq (%)
**6-1**	**CASLAGVYEQFF**	**2-1**	**45**	**10.6**
19	CASSIRAPRPEQFF	2-1	20	4.7
**30**	**CAWSEQAYTEAFF**	**1-1**	**20**	**4.7**
5-5	CASGDGSTDTQYF	2-3	16	3.8
7-3	CASSATGRMSEAFF	1-1	15	3.5
29-1	CSVDTGGGQPQHF	1-5	14	3.3
6-1	CASSELGRSYEQYF	2-7	13	3.1
7-2	CASSPPGGNTEAFF	1-1	12	2.8
25-1	CASSEYRATEAFF	1-1	12	2.8
11-3	CASSSLWGNEQFF	2-1	12	2.8
12-4	CASSLQGSYNSPLHF	1-6	10	2.4
29-1	CSVGGHSNQPQHF	1-5	10	2.4
11-2	CASSDGTEATYEQYF	2-7	9	2.1
14	CASSQVTGVNTEAFF	1-1	8	1.9
2	CASSKGAGANVLTF	2-6	8	1.9

Figure S3. High-throughput analysis of expressed *TRB* gene rearrangements. The top 15 most frequent clonotypes are listed for the indicated subsets. Sequences were derived from RNA-Seq data using MiXCR. Clonotypes appearing in bold represent sequences found within the CCR8^{+} and CCR8^{-} populations.
